# The Extrastriate Body Area and identity processing: An fMRI guided TMS study

**DOI:** 10.14814/phy2.14711

**Published:** 2021-05-02

**Authors:** Alizée Pann, Mireille Bonnard, Olivier Felician, Patricia Romaiguère

**Affiliations:** ^1^ Aix Marseille Univ INSERM, INS, Inst Neurosc Syst Marseille France; ^2^ Aix Marseille Univ, APHM, INS, Hôpital de la Timone Service de Neurologie et de Neuropsychologie Marseille France; ^3^ Aix Marseille Univ CNRS, ISM Marseille France

**Keywords:** extrastriate body area, functional MRI, TMS, visual perception

## Abstract

The extrastriate body area (EBA) is a body‐selective focal region located in the lateral occipito‐temporal cortex that responds strongly to images of human bodies and body parts in comparison with other classes of stimuli. Whether EBA contributes also to the body recognition of self versus others remains in debate. We investigated whether EBA contributes to self‐other distinction and whether there might be a hemispheric‐side specificity to that contribution using double‐pulse transcranial magnetic stimulation (TMS) in right‐handed participants. Prior to the TMS experiment, all participants underwent an fMRI localizer task to determine individual EBA location. TMS was then applied over either right EBA, left EBA or vertex, while participants performed an identification task in which images of self or others' right, or left hands were presented. TMS over both EBAs slowed responses, with no identity‐specific effect. However, TMS applied over right EBA induced significantly more errors on other's hands than noTMS, TMS over left EBA or over the Vertex, when applied at 100–110 ms after image onset. The last three conditions did not differ, nor was there any difference for self‐hands. These findings suggest that EBA participates in self/other discrimination.

## Graphical abstract

The present study provides evidence that EBA in the right hemisphere participates in identity processing. TMS over the right EBA might disrupt the early processing of visual signals that would be important in an explicit recognition task, leading to decreased response speed and accuracy.

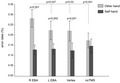

## 1 INTRODUCTION

The Extrastriate Body Area (EBA) is a functional region located in the lateral occipito‐temporal cortex (Downing et al., 2001). In functional MRI studies, viewing body representations elicits higher responses in EBA than other stimuli, suggesting a specific role in the visual perception of bodies. EBA has also been investigated using transcranial magnetic stimulation (TMS), which provides a complementary approach by producing transient and reversible interference with the activity of the targeted region. Early studies confirmed the implication of EBA in body perception, showing that repetitive TMS (rTMS) over EBA disrupted performance in the visual processing of non‐facial body parts, while leaving unchanged visual processing of face parts and non‐corporeal stimuli (Pitcher et al., 2009; Urgesi et al., 2004). In addition, TMS interfered with the processing of inverted but not upright bodies (Urgesi et al., 2007), suggesting that EBA processes isolated body parts rather than whole bodies.

Beyond the visual perception of bodies and body parts, several lines of evidence suggest that EBA represents the body in a multisensory and dynamic manner (Jeannerod, 2004). For instance, fMRI studies have found a significant activation of EBA while viewing moving bodies (Di Vita et al., 2016; Downing et al., 2006; Felician et al., 2009), but also during pointing movements performed without the vision of the acting body part (Astafiev et al., 2004) and preparation of manual action (Kühn et al., 2011), suggesting that EBA receives visual and sensory‐motor signals of the acting body.

In addition to the visual perception of bodies and the control of voluntary actions, the possibility that EBA could play a role in processing identity has also been raised. In an fMRI adaptation study, Myers and Sowden (2008) found a greater adaptation in blocks only comprising views of other hands, as compared to blocks comprising views of both self and other hands. This adaptation effect was more pronounced in the right hemisphere. Right EBA also responded higher when participants looked at their own body than at another body (Vocks et al., 2010). In contrast, rEBA activation was found stronger in response to images of body parts presented from an allocentric rather than from an egocentric perspective, while no viewpoint‐dependent difference was observed in the lEBA (Saxe et al., 2006). These findings were also consistent with a role of rEBA in body identity, but more pronounced when processing other's body parts than one's own. However, Chan et al. (2004) tested whether EBA distinguished between egocentric and allocentric views of the self and other bodies. While they also found increased rEBA activity when viewing allocentric relative to egocentric views, identity had no effect in either EBA. In addition, Hodzic, Kaas, et al. (2009) and Hodzic, Muckli, et al. (2009) found no modulation of rEBA activations in distinguishing self‐body from the bodies of familiar others.

Diverging findings regarding left EBA (lEBA) have also been reported. Hodzic, Kaas, et al. (2009) and Hodzic, Muckli, et al. (2009) found a modulation of the lEBA when contrasting self and others' body images, with a stronger response to self than to other bodies. However, these findings were recently challenged in a TMS experiment relying on the visual enhancement of touch paradigm (vision of the body enhances spatial tactile acuity on the seen body part even if the tactile stimulation is invisible; Beck et al., 2015). TMS over lEBA attenuated the visual enhancement of touch both when participants observed their own hand and another person's hand, suggesting that lEBA participates in a common visual representation of the human body with no regard to ownership or identity.

Taken together, studies focusing on the role of EBA in discriminating one's own body parts from others have yielded conflicting results, leaving this issue unresolved. In a previous paper, we speculated that both right and left EBA provide input into the motor system, although with distinct roles in action representation. Left EBA would be part of a network involved in action understanding, while right EBA would be part of a network involved in the processing of actions with the aim to disentangle those produced by oneself from others and experience the sense of agency (Romaiguère et al., 2014). This entails that right EBA would be involved in self/other discrimination, but not left EBA. The aim of the present study is to test this hypothesis.

EBA involvement in identity processing may be addressed through implicit or explicit recognition tasks. Here an explicit recognition task was used in order to focus participants' attention on identity. Participants were thus requested to view photographs of right and left hands and determine whether the hand belonged to them or not. While they were performing the task, we applied paired‐pulse TMS over either left or right EBA, or over the vertex as a control site for TMS effects. Another crucial parameter for TMS is the time of application relative to the task. We referred to a study by Pitcher et al. (2012) who documented two distinct time windows of EBA activity, an early non‐category‐specific stage, occurring at 40/50 ms following stimulus onset, and a later category‐specific (i.e., specific to the body part processing) stage, occurring at 100/110 ms after stimulus onset. Based on their results, one could expect identity specificity to be more likely to occur during the body part specific stage, but as self‐attribution is a very important process, one cannot rule out that self‐specificity could occur during the earlier stage. Both delays were, therefore, tested. According to our hypothesis, we expected TMS over right EBA to impair self‐other discrimination, but not TMS over left EBA. It has to be noted that the delay of TMS application is irrelevant for that main hypothesis.

## 2 MATERIALS AND METHODS

### 2.1 Participants

Based on a study of functional accuracy of TMS in cognitive studies, (Sack et al., 2009), and on the literature, sixteen participants were recruited and entered the TMS sessions. They were healthy and right‐handed (mean age 31 ± 6.3 years; 4 males, 12 females). They gave their written informed consent to participate in the study, completed a safety screening questionnaire for fMRI and TMS and were financially compensated. All of them were neurologically healthy, had normal or corrected‐to‐normal vision, were informed about the procedure, and were naïve to the purpose of the experiment. Participants first completed the fMRI session, then received on a different day the TMS session, that is, three runs targeting the three different sites and the no‐TMS run. The procedure was approved by the local ethics committee (CPP Sud‐Méditerranée I) and was in accordance with the declaration of Helsinki.

### 2.2 fMRI localizer acquisition

#### 2.2.1 MRI scanner and scanning parameters

Each participant underwent an fMRI localizer to identify functional regions of interest with respect to individual brain anatomy. Scanning was performed using a 3T whole‐body imager MEDSPEC 30/80 AVANCE (Bruker, Ettlingen, Germany) equipped with a circularly polarized head coil. High‐resolution structural T1‐weighted images were acquired from all participants for anatomical localization. The anatomical slices covered the whole brain and were acquired parallel to the sagittal plane. The functional images were acquired using a T2*‐weighted echo‐planar sequence with 36 axial slices (repetition time = 2.4 s, interleaved acquisition, slice thickness: 3 mm, field of view = 19.2 × 19.2 cm, 64 × 64 matrix of 3 × 3 mm voxels. The slices were parallel to the AC–PC plane and covered the whole brain. Participants were studied in one functional run of 247 scans, with a total duration of 9.9 min. For each run, the scanner was in the acquisition mode for 12 s before the experiment to achieve the steady‐state transverse magnetization.

#### 2.2.2 Experimental design

**FIGURE 1:**
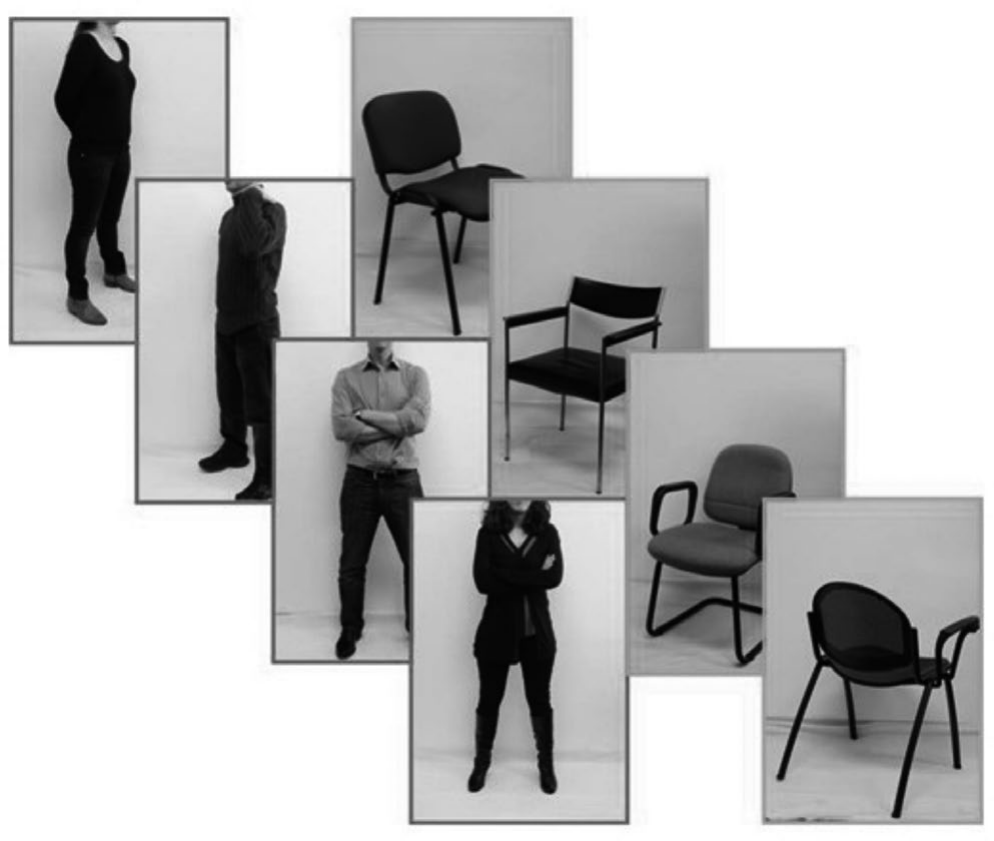
Blocks of images (640 × 480 pixels of size) of human bodies versus chairs, in different positions, used for the fMRI localizer. The Bodies and Chairs condition were presented in 12 s blocks. In each bodies or chair block, 12 images from one category were presented 800 ms and segregated by a 200 ms black screen. Once or twice during each stimulus block, the same pose/position was presented twice in succession. Participants were instructed to detect these immediate repetitions and report them with a button press (1‐back task).

We were interested in EBA's function, we, therefore, performed a classical EBA localizing contrast: headless bodies versus chairs. Moreover, it was essential not to include hands in the localizer because there is an area in the left lateral occipito‐temporal cortex that specifically responds to hands (Bracci et al., 2010). However, this area has been shown to be anatomically different from EBA, and it seems to respond to hands more as functional effectors rather than as anatomical body parts (Bracci et al., 2010). As shown in Figure 1, stimuli consisted of black and white photographs (640 × 480 pixels of size). They were delivered to a high luminance LCD projector, back‐projected onto a frosted screen positioned at the back end of the MRI tunnel, and viewed by the participants through a mirror.

The EBA localizer consisted of 12s blocks of images of human bodies (without heads) in different poses, 12s blocks of images of chairs in different positions, and 12s resting blocks with a fixation cross. The three conditions were presented in a pseudo‐random order, with each condition never presented more than twice consecutively. The bodies and chairs conditions were presented 18 times each during the scan, while the fixation condition was presented nine times, resulting in 247 fMRI volumes. In each bodies or chair block, 12 images from one category were presented 800 ms and segregated by a 200 ms black screen. All images appeared against a white background. Once or twice during each stimulus block, the same pose/position was presented twice in succession. Participants were instructed to detect these immediate repetitions and report them with a button press (1‐back task). Stimulus presentation was controlled by the LabVIEW software package (National Instruments Corporation, Austin, TX, USA).

#### 2.2.3 Image preprocessing and first level statistical analysis

Standard data preprocessing and statistical analysis were performed using the Statistical Parametric Mapping package (SPM12, Wellcome Department of Cognitive Neurology, London). Preprocessing steps consisted of slice timing correction, realignment to the mean of the images to correct for motion, coregistration of anatomical images to the functional images, and subsequent reslicing. The functional data were then spatially smoothed (6 × 6 × 6 mm). For each participant, a general linear model was applied to the time course of the functional signal at each voxel. Each condition was modeled by a 12s box‐car function synchronized with the individual trials of this condition and convolved with a canonical hemodynamic response function. In each participant, statistic parametric maps were calculated for individual T‐contrasts Bodies versus Chairs. The results were reviewed with the threshold of significance for active voxel set at *p* < 0.001 uncorrected.

#### 2.2.4 ROI definition

**FIGURE 2:**
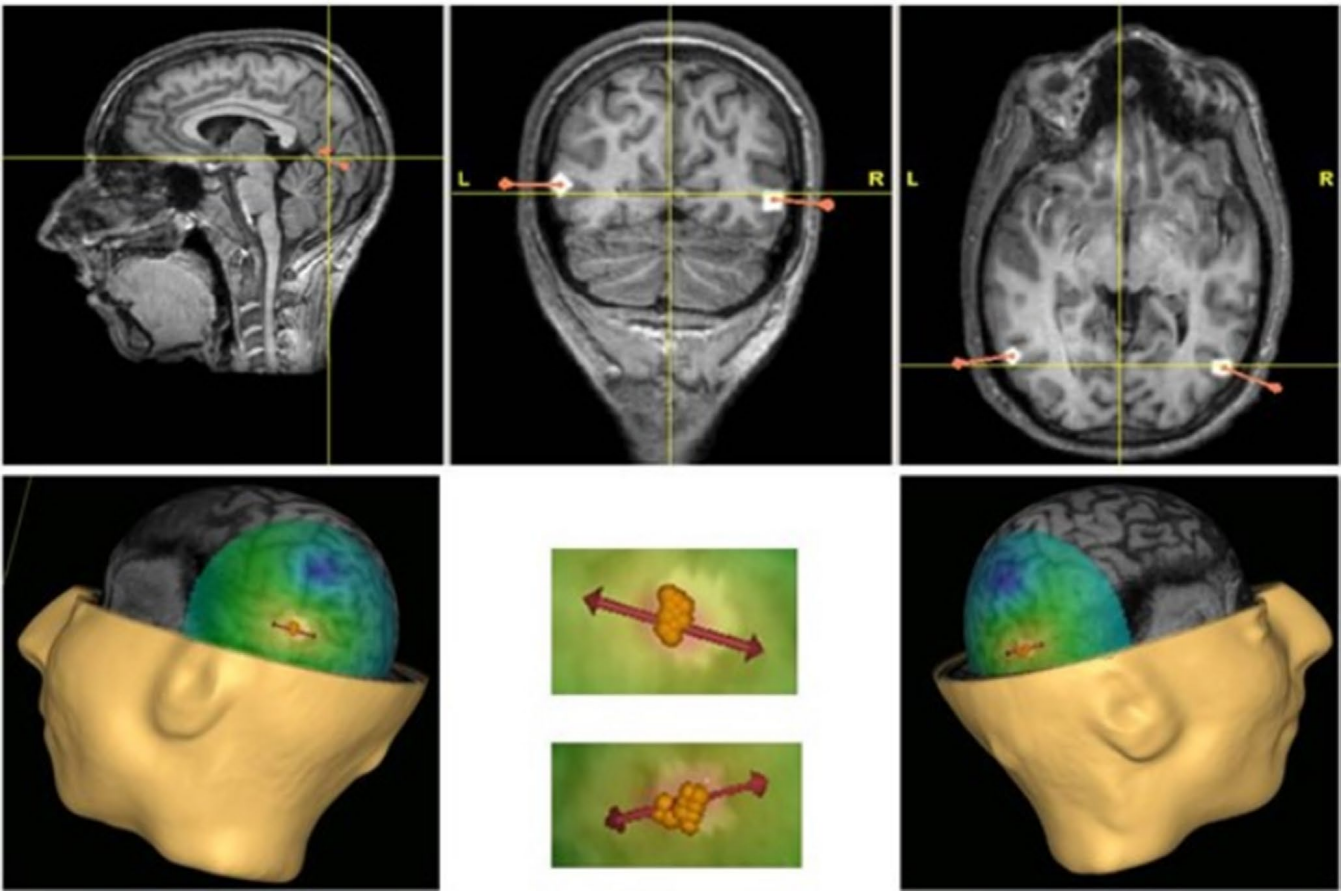
Top row: Region of interest identified for this subject in both left and right hemispheres. Bottom row: 3D representation of the defined rEBA and lEBA targets represented by the white points on the anatomical image (left and right images) and superposition of the markers (yellow dots) placed by the neuronavigation software on the anatomical image at each TMS pulse for both rEBA and lEBA (central image). The superposition of all successive pulses shows the accuracy and reproducibility of TMS parameters. Regions of interest (ROIs) were defined in individual participants using the data acquired from the EBA localizer scan. The most significant voxel in the occipital‐temporal region (peak EBA voxel) was identified in each hemisphere. The EBA ROIs were constructed by setting a 1 cm diameter sphere centered on the peak EBA voxel to ensure that an area of maximal response to body images was localized, while minimizing the inclusion of other functional areas.

Regions of interest (ROIs) were defined in individual participants using the data acquired from the EBA localizer scan. Left and right EBA were localized in each participant by a body minus chair contrast. The most significant voxel in the occipital‐temporal region (peak EBA voxel) was identified in each hemisphere. The EBA ROIs were established by setting a 1 cm diameter sphere centered on the peak EBA voxel to ensure that an area of maximal response to body images was localized while minimizing the inclusion of other functional areas (Figure 2a).

### 2.3 TMS session

#### 2.3.1 Materials and stimulation procedures

**FIGURE 3:**
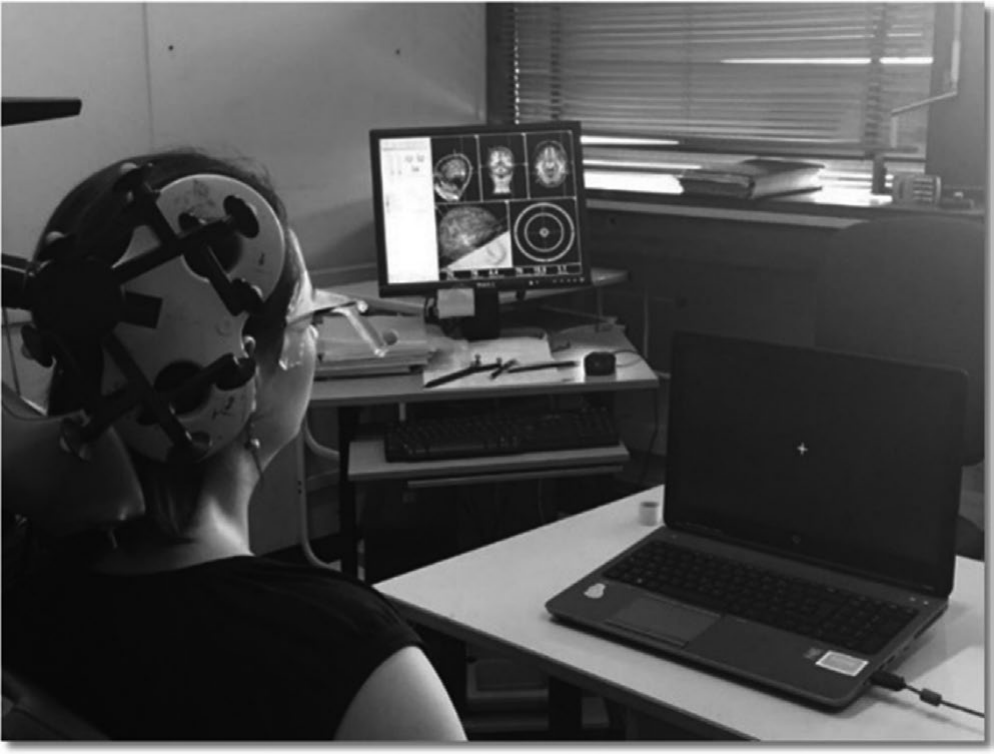
Participant's set‐up, coil, and neuronavigation monitoring. Participants were comfortably seated in an armchair, facing the computer screen. The coil was firmly held in place with mechanical arms and its location relative to the EBA target was continuously monitored with neuronavigation. The neuronavigation system allowed to accurately and reproducibly maintain stimulation targets.

Participants were comfortably seated in an armchair, facing the computer screen (Figure 3). The coil was firmly held in place with mechanical arms and its location relative to the EBA target was continuously monitored with neuronavigation (Navigation Brain System, Nexstim 2.3, Helsinki, Finland); it was adjusted when needed by the experimenter. The neuronavigation system allowed to accurately and reproducibly maintain stimulation targets (Figure 2c). During the TMS session, stereotaxic coordinates for the localization of the successive TMS stimulations were recorded (Figure 3).

Stimulation was performed using two Magstim 200 stimulators coupled with a bi‐stim module (Magstim, Whitland, UK) and a coplanar figure‐of‐eight coil with an external loop diameter of 9 cm. The stimulation intensity was adjusted for each participant and for each ROI. For each participant, the hand area of the primary motor cortex in the left hemisphere (M1), was first identified anatomically according to the method of Yousry et al. (1997). It was then stimulated with increasing TMS intensity until a twitch of the right hand's fingers was visible. This TMS intensity, necessary to evoke a peripheral motor response, corresponds to the resting motor threshold (RMT) and is variable across individuals. The value of the electric field evoked in M1 by TMS at 110% RMT, calculated by the neuronavigation software, was noted. Then for each ROI, we adjusted the stimulation intensity so that the electric field value in each ROI (left EBA, right EBA or Vertex) corresponded to the electric field value induced in M1 by TMS at 110% RMT. Table 1 gives the stimulation intensity and the corresponding electric field values for each ROI (L‐EBA, R‐EBA, Vertex) individually. Stimulation parameters were well within international safety guidelines (Rossi et al., 2009; Wassermann, 1998).

**TABLE 1:** TMS specifications. Intensity: Magnetic stimulator output (% of the maximum output). EF: Electric field at target (V/m), x,y,z: Coordinates of the ROIs (MNI referential). ROIs were constructed in the subject's space (non‐normalized data) to accurately guide the TMS. Left and right EBA coordinates were normalized into MNI referential for the publication only, to allow comparison with other studies. There are no MNI coordinates for the Vertex ROIs. The vertex was identified on the basis of cranial measures on each participant, not on the basis of a functional localizer like EBA.

	LEBA			REBA			Vertex	
	x,y,z	Intensity	EF	x,y,z	Intensity	EF	Intensity	EF
P1	−51, −72, 15	38	56.86	51, −66, 0	31	61.07	31	67.31
P2	−54, −69, 6	37	61.23	51, −66, 3	30	59.5	32	59.38
P3	−51, −72, 9	37	74.89	51, −69, 0	32	78.58	45	49.3
P4	−48, −75, 12	34	74.65	60, −48, −3	32	70.19	40	68.51
P5	−48, −81, 15	35	73.31	57, −66, 9	42	71.16	33	69.14
P6	−51, −75, 9	36	60.56	51, −72, 6	33	47.55	35	52.08
P8	−48, −72, 18	37	64.61	45, −72, 12	40	68.95	36	65.8
P9	−48, −75, 6	31	61.84	45,−72, 9	31	63.57	37	63.19
P12	−57, −63, 9	37	66.83	54, −60, 12	38	64.07	40	53.38
P13	−45, −69, 3	30	61.23	51, −63, 15	36	59.59	45	24.58
P14	−51, −72, 3	38	56.18	45, −72, 15	32	56.63	42	39.18
P16	−48, −75, −3	30	66.82	48, −66, 6	35	50.58	44	50.5
Mean		35	64.92		34.33	62.62	38.33	55.20
Std		3.05	6.53		3.89	8.83	5.05	13.40

We chose paired‐pulse TMS because the summation properties of TMS pulses offer longer inactivation period than single‐pulse TMS but still allow good temporal resolution defined by the temporal distance between the two pulses (Silvanto et al., 2005; Walsh & Pascual‐Leone, 2003). According to Pitcher et al.'s (2012) results, we chose to deliver paired‐pulse stimulation during the task (image discrimination) at two time‐windows: 40 and 50 ms post‐visual stimulus onset (40–50 ms), or 100 and 110 post‐visual stimulus onset (100–110 ms).

#### 2.3.2 Experimental design

**FIGURE 4:**
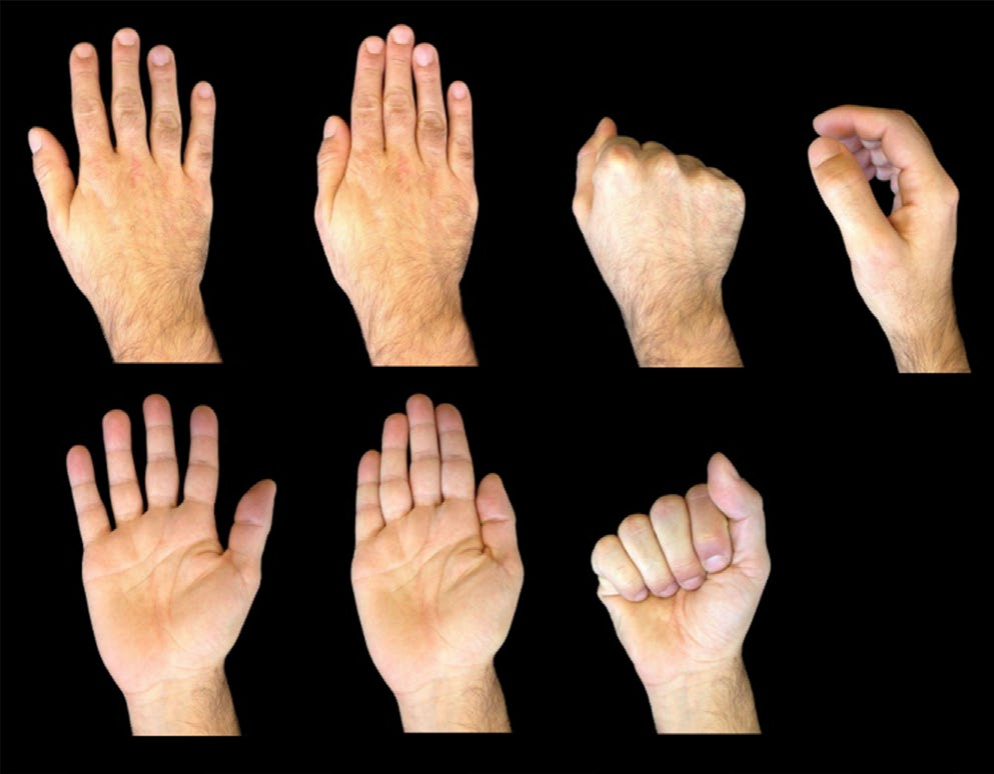
Examples of stimuli used in the TMS study, with the seven hand positions. There were two visual stimuli conditions: Self Hand and Other Hand. Each condition corresponded to 14 images: seven different positions each for the right hand and the left hand. Photographs were taken above a black background after jewelry and/or nail polish were removed. Sleeves were pulled up so they would not show on the photographs. The images were resized to 480 × 640 pixels.

There were two visual stimuli conditions: Self Hand and Other Hand. Each condition corresponded to 14 images: seven different positions each for the right hand and the left hand (Figure 4). Photographs were taken above a black background after jewelry and/or nail polish were removed. Sleeves were pulled up so they would not show on the photographs. Participants were shown the position they had to produce before each photograph. The images were resized to 480 × 640 pixels.

Each session contained a total of four acquisition runs corresponding to TMS over each of the two ROIs (right EBA, left EBA), the control site (Vertex), and a no‐TMS run. Each run contained an identical number of Self Hand and Other Hand trials. To homogenize conditions, Other Hand trials contained images of the right and left hand of a single individual of the same gender than the participant. Stimuli were delivered during 200 ms, in a pseudo‐random order, with a 5000 ms inter‐stimuli black screen and each stimulus was preceded by a 1000 ms fixation cross. When applied, double‐pulse TMS occurred at either one of the two time‐windows: 40 and 50 ms post‐visual stimulus onset (40–50 ms), or 100 and 110 ms post‐visual stimulus onset (100–110 ms). Presentation, timing, TMS triggering, and data collection were controlled by the LabVIEW software package.

Participants performed a training run, consisting of 20 trials to get acquainted with the images and with the response pad. After that, the no‐TMS run was always the first run, to serve as an initial control condition to observe the task performance independently of the application of numerous TMS pulses, practice/learning effect, attentional fatigue throughout the experiment. Then, we had two runs corresponding to TMS over left or right EBA, our main regions of interest), the order of which was counterbalanced across participants. Finally, we had the last run during which TMS was applied over the vertex (not involved in the discrimination task), to serve as a final control condition to verify that the potentially observed effects in the EBA runs are specific to EBA stimulation and not to nonspecific effects due to the stimulation of any brain area. We did not counterbalance the Vertex run with the EBA runs (which might have been a better control for the effect of TMS), indeed, by running the Vertex run last and comparing it with the No‐TMS run, we could verify that there was no effect of practice/learning in the experimental runs. It also allowed to control for effects of successive TMS pulses, or attentional fatigue, on task performance throughout the experiment.

During each run, participants were required to focus their attention on the screen. They were instructed to respond as fast as possible by pressing one of the two pushbuttons of the response box (with their right index finger or with their right middle finger) to indicate whether the hands presented on the screen belonged to them or not. The association between response and finger was counterbalanced across participants: for half of them the right push‐button corresponded to the answer “this is mine,” while for the other half the right button corresponded to the answer “this is not mine.” Accuracy and reaction times (RTs) were recorded.

#### 2.3.3 Statistical analyses

There were two image types (Self Hand and Other Hand), four TMS settings (Right EBA, Left EBA, Vertex, and noTMS), and two TMS time windows (40–50 ms and 100–110 ms), resulting in 14 conditions. Individual mean error rates and reaction times (RTs) were calculated for each condition independently and subjected to separate ANOVAs. We did not have a full cross‐over design, as noTMS does not have time windows. We, therefore, ran separate ANOVAs for each delay (40–50 ms, 100–110 ms), using the Statistica software (StatSoft Inc.). All four ANOVAs were repeated‐measures 2‐way ANOVAS with a factor Owner with two levels (Self or Other) and a factor TMS with four levels (Right EBA, Left EBA, Vertex, noTMS). The ANOVA plan was thus: TMS [rEBA, lEBA, Vertex, noTMS] x Owner [Self, Other]. Because we ran separate ANOVAs for the two time‐windows, we applied a Bonferroni correction for multiple comparisons. The significance level for the main effects and interactions of each ANOVA was thus set at 0.025. When needed, post hoc analyses were performed using Fisher's Least Square Difference method, with a significance level set at 0.05, as these were performed within the ANOVAs.

## 3 RESULTS

### 3.1 fMRI localizer

EBA was identified in both hemispheres in all participants. For the TMS experiment, ROIs were built, for each participant around coordinates in the individual subject's space (non‐normalized data), and cannot be compared with data available in the literature, that are normalized in either Talairach or Montreal Neurological Institute (MNI) space. Therefore, we also normalized the data and ran a random‐effects group analysis. Right EBA was found as a cluster of 53 voxels (*p* < 0.001, FWE corrected at cluster level) with a peak voxel at 51 ‐63 12 (MNI referential; T value = 10.02), and left EBA was found as a cluster of 100 voxels (*p* < 0.001, FWE corrected at cluster level) with a peak voxel at ‐51 ‐69 15 (T value = 8.39). Both clusters were localized at the junction between middle temporal and middle occipital gyri using the Anatomy toolbox (Eickhoff et al., 2005, 2006, 2007) and the Anatomical Automatic Labeling toolbox (Tzourio‐Mazoyer et al., 2002).

### 3.2 TMS experiment

As our analysis relies on the comparison of responses for Self Hands and Other Hands, we computed error rates independently for each condition. Error rates were compared to chance (50%) using a binomial test, for the noTMS run only, as TMS could induce higher error rates. Data from four participants were discarded based on those tests. Three participants had error rates that were not different from chance for Self Hands or for Other Hands in the noTMS run, meaning they did not truly discriminate self from other hands, even without TMS. One participant had error rates significantly superior to 50% in the noTMS run, meaning they had a response bias, even before TMS. The results of the binomial tests are given in Table 2 for the four participants excluded. For the remaining 12 participants, error rates in the noTMS condition were 17.38% ± 3.63% for Other Hands and 14.67% ± 3.46% for Self Hands, and mean error rates in the TMS runs were 21% ± 3.3% for Other Hands and 13% ± 2.9% for Self Hands (mean ± SE). Results for those participants (mean age 29.9 ± 5.24 years; 2 males, 10 females) are detailed below.

**TABLE 2:** Results of the binomial test for the four participants excluded from the analyses. Error rates during the noTMS condition were compared to chance (50%) using a binomial test. Proper performance of the task corresponds to error rates significantly lower than 50%. P10, P11, and P15: performance non‐significantly different from chance for Self Hand or Other Hand. P7: error rates for Other Hand significantly greater than 50% (response bias).

	noTMS		
	Error rate	Z	*p*
P7			
Self hand	1.79%	−7.08	<0.0001
Other hand	80.36%	4.41	<0.001
P10			
Self hand	35.71%	−2.00	0.023
Other hand	42.86%	−0.94	0.17
P11			
Self hand	42.86%	−0.94	0.17
Other hand	25.45%	−3.51	0.00023
P15			
Self hand	33.93%	−2.27	0.012
Other hand	58.93%	1.20	0.11

#### 3.2.1 Error rates

Error rates were calculated as nb of errors/number of trials for each condition.

When TMS was applied 40–50 ms after image onset, there were no significant main effects or interaction.

**FIGURE 5:**
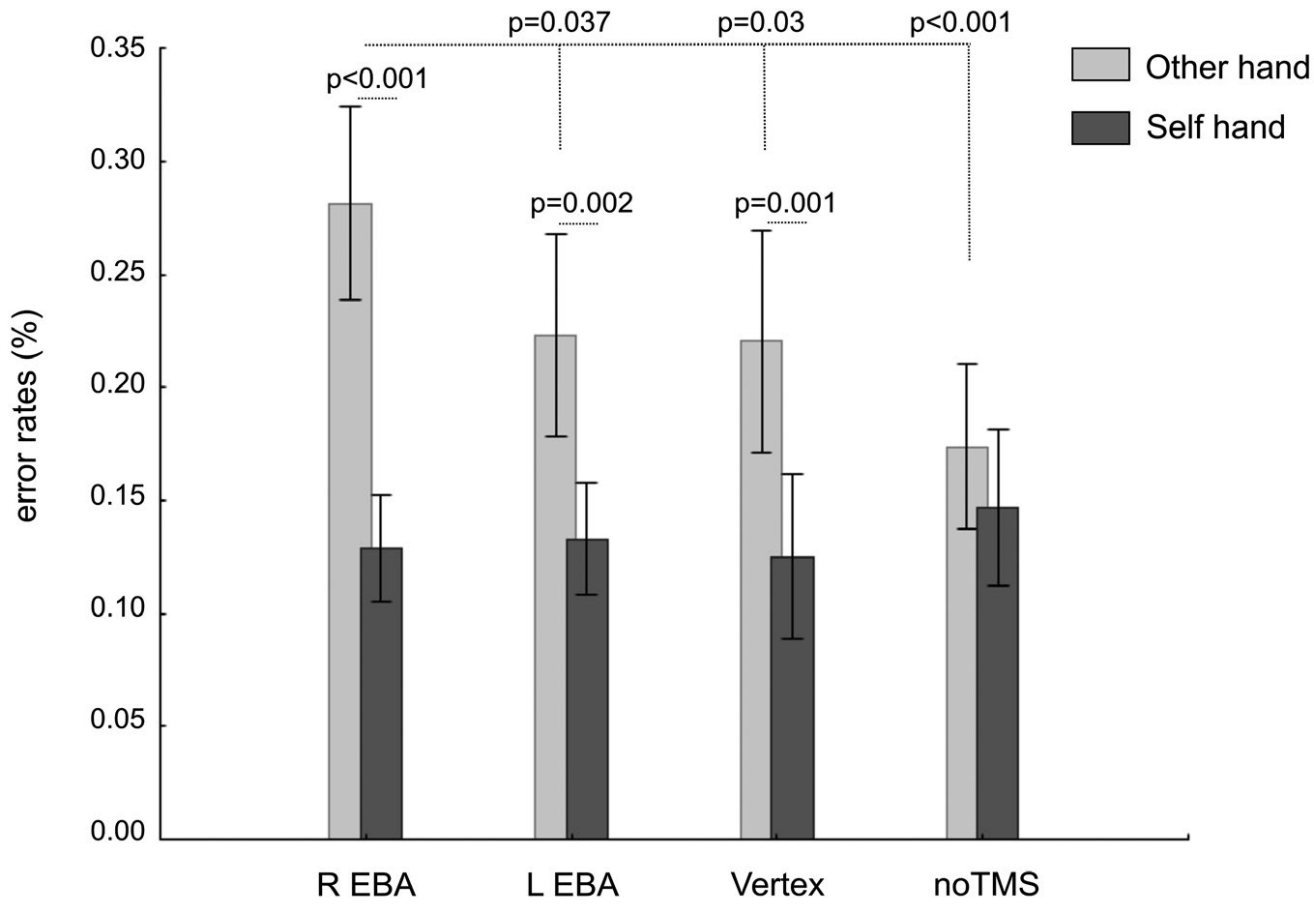
Error rate (nb of errors/number of trials) for other's hands (OH) and self hands (SH) when TMS pulses were targeted to rEBA, lEBA, Vertex, and during the noTMS condition at the 100–110 ms time‐window. Values are mean ± SE, n = 12. For clarity, only significant differences are indicated with their respective p values. There was an interaction between the factors TMS and Owner (*F*_3,33_ = 3.659, *p* = 0.022). Post hoc analyses on the TMS x Owner interaction showed that TMS over Right EBA induced significantly more errors on Other Hands than all three other conditions (significance values are shown on the graph). Error rates for Other Hand were not different between Left EBA, Vertex, and noTMS. Error rates for Self Hand were not different between the four conditions.

When TMS was applied 100–110 ms after image onset there was an interaction between the two factors (*F*_3,33_ = 3.659, *p* = 0.022, η_p_^2^ = 0.25). Post hoc analyses on the TMS x Owner interaction showed that TMS over Right EBA induced significantly more errors on Other Hands than all three other conditions (Figure 5). Moreover, error rates for Other Hand were not different between Left EBA, Vertex, and noTMS. Error rates for Self Hand were not different between the four conditions. However, TMS increased the difference in error rates between Self Hand and Other Hand on all three targets, resulting in an effect of the factor Owner (*F*_1,11_ = 6.689, *p* = 0.025, η_p_^2^ = 0.38), such that there were more errors for Other Hands than for Self Hands.

#### 3.2.2 Reaction times

When TMS was applied 40–50 ms after image onset, there was a main effect of the factor TMS (*F*_3,33_ = 6.43; *p* = 0.0015, η_p_^2^ = 0.37), and of the factor Owner (*F*_1,11_ = 6.67; *p* = 0.025, η_p_^2^ = 0.38), such that reaction times for Other Hands were longer than for Self Hands. There was no interaction between the factors (*F*_3,33_ = 1.97, NS, η_p_^2^ = 0.15).

**FIGURE 6:**
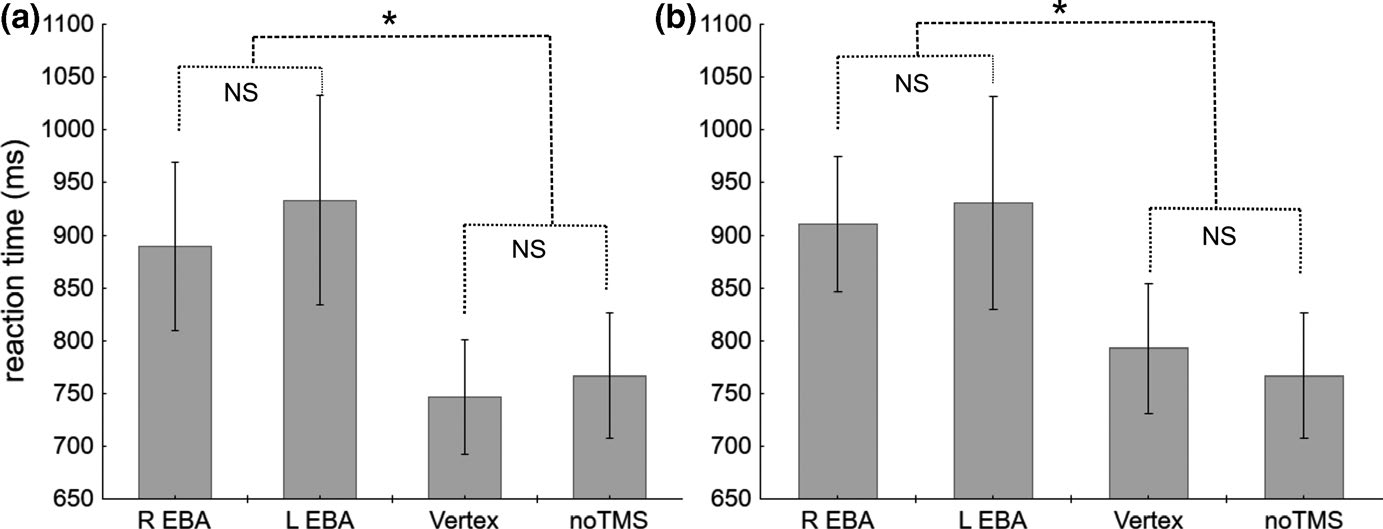
Reaction times for hand discrimination when TMS pulses were targeted to rEBA, lEBA, Vertex, and during the No‐TMS condition at the 40–50 ms (a) and the 100–110 ms (b) time‐windows. Values are mean ± SE, n = 12. NS: not significant, *: significant difference. There was no interaction between the factors TMS and Owner at either delay; therefore, Self Hands and Other Hands data could not be analyzed separately. There was a main effect of the factor TMS at both delays (*F*_3,33_ = 6.43; *p* = 0.0015, at 40–50 ms and *F*_3,33_ = 6.21; *p* = 0.002 at 100–110 ms). Post hoc analyses on the TMS factor showed similar results at both delays: TMS over Right EBA and Left EBA slowed reaction times compared to the noTMS condition and the Vertex condition. The Right and Left EBA conditions were not significantly different from each other (*p* = 0.40 at 40–50 ms and *p* = 0.67 at 100–110 ms), neither were the Vertex and noTMS conditions (*p* = 0.69 at 40–50 ms and *p* = 0.58 at 100–110 ms).

Post hoc analyses on the TMS factor (Figure 6a) showed that TMS over Right EBA (889.41 ms ± 79.97 ms, mean ± SE) and Left EBA (933.18 ms ± 99.29 ms) slowed reaction times compared to the noTMS condition (766.83 ms ± 59.42 ms, both *p* < 0.05) and the Vertex condition (746.56 ms ± 53.78 ms, both *p* < 0.05). The Right and Left EBA conditions were not significantly different from each other (*p* = 0.40), neither were the Vertex and noTMS conditions (*p* = 0.69).

When TMS was applied 100–110 ms after image onset, there was also a main effect of the factor TMS (*F*_3,33_ = 6.21; *p* = 0.002, η_p_^2^ = 0.36), but no main effect of the factor Owner (*F*_1,11_ = 3.49; *p* = 0.089, η_p_^2^ = 0.24). There was no interaction between the factors (*F*_3,33_ = 0.247, NS, η_p_^2^ = 0.02).

Post hoc analyses on the TMS factor (figure 6b) showed that TMS over Right EBA (910.41 ± 64.08) and Left EBA (930.62 ± 100.92) slowed reaction times compared to the noTMS condition (766.83 ± 59.42, both *p* < 0.05) and the Vertex condition (792.84 ± 61.57, both *p* < 0.05). The Right and Left EBA conditions were not significantly different from each other (*p* = 0.67), neither were the Vertex and noTMS conditions (*p* = 0.58).

## 4 DISCUSSION

According to the literature, EBA is involved in the perception of bodies and non‐facial body parts (Downing et al., 2001; Pitcher et al., 2009; Taylor et al., 2007; Urgesi et al., 2004, 2007), but its role in disentangling body identity remains unclear. Here we used double‐pulse transcranial magnetic stimulation to test the role of EBA in self‐other distinction and the possibility of the functional specialization of right and left EBA. Prior to the TMS experiment, all participants underwent an fMRI localizer task to determine targets' location precisely. Double‐pulse TMS was then applied over either right EBA, left EBA or the vertex (control condition), while participants performed a discrimination task on a computer screen. They had to determine as soon as possible whether the hand presented was theirs or not. TMS applied over the vertex had no effect on error rates or RTs compared to the no‐TMS condition at either TMS time window. TMS over either EBA slowed RTs at both time windows. Moreover, TMS over the right EBA at 100–110 ms induced more errors on other hands than any other condition, including TMS over the left EBA. TMS over left EBA did not impact error rates, which does not totally exclude the possibility that left EBA is involved in identity processing. Indeed, our statistical analyses are based on a relatively low number of participants and might have lacked the power to evidence weak effects.

Past studies have suggested that some of the effects reported in TMS studies could be due to TMS side effects rather than to the disruption of cerebral function (Abler et al., 2005; Duecker et al., 2013; Holmes & Meteyard, 2018; Meteyard & Holmes, 2018). These side effects include noise, cutaneous stimulation, and possible twitches in head/face muscles that cause subjective discomfort that has been shown to affect task performance (Meteyard & Holmes, 2018). These authors have found that subjective discomfort differs as a function of TMS target and possibly of TMS intensity. Importantly, TMS over the vertex elicited less discomfort than most other sites, leading the authors to question the validity of the vertex as a control condition. The question thus arises whether the different effects reported here could be explained by TMS side effects and/or subjective discomfort. While these effects could explain the differences observed between Vertex TMS and EBAs TMS, side effects and discomfort are less different between left and right EBA, thus less likely to explain the difference observed with TMS over the two sites. Also, these factors could explain an increased number of errors in the task but seem less likely to explain why only errors on Other hands increased, not errors on Self hands that did not differ across all four conditions (noTMS, Vertex, Right EBA, Left EBA). Moreover, the intensities used in the present studies were relatively low (between 30 and 40% of the magnetic stimulator output), lower than in the studies of the effects of TMS side effects (Duecker et al., 2013; Meteyard & Holmes, 2018). Meteyard and Holmes suggested that TMS intensity could be of importance in subjective discomfort. From their supplementary data, there seems to be no to little discomfort for the intensities we used (Meteyard & Holmes, 2018). Finally, Duecker et al. showed that TMS timing was also of importance in the interference between TMS side effects and task performance. For delays between stimulus onset and TMS comprised between 0 and 100 ms like ours, there was no effect on accuracy nor on reaction times for both tasks studied (Duecker et al., 2013). On the other hand, the slowing of reaction times when TMS is applied over either EBA is consistent with previous studies about the role of EBA as a specialized structure involved in human body and body part processing (Downing et al., 2001; Myers & Sowden, 2008; Saxe et al., 2006; Taylor et al., 2007; Urgesi, 2007). Indeed, the results of Downing et al. (2001) described EBA as a region of the human lateral occipitotemporal cortex selectively responding to visual images of human bodies and body parts, with the exception of faces. Following the lead of Downing et al. (2001), many other neuroimaging studies have found the same effect and support the notion that EBA is selective for images of the body and body parts relative to a variety of control images (Orlov et al., 2010; Peelen & Downing, 2007; Pinsk et al., 2009; Schwarzlose et al., 2008; Spiridon et al., 2006). These results support the notion that TMS effects reported here can more readily be explained by cortical effects of TMS, rather than by TMS side effects and/or subjective discomfort.

That these effects are likely cortical in origin does not entail that they are specific of body processing being disrupted by TMS. Indeed, we did not use an object control condition, so we cannot assert that we would not have found the same effects had we presented different types of stimuli. However, TMS over EBA has been shown to be specific to body stimuli. For example, TMS over EBA impairs the visual processing of non‐facial body parts, but not that of faces or other objects (Pitcher et al., 2009; Urgesi et al., 2004). It also impairs the detection of people in natural scenes but not that of cars (van Koningsbruggen, 2013). Beyond being category‐specific, all those effects are also specific to TMS over the EBA when compared with neighboring areas such as the primary visual cortex (V1, Urgesi et al., 2004), the Occipital Face Area, and the Lateral Occipital complex (Pitcher et al., 2009) and the Transverse Occipital Sulcus (van Koningsbruggen, 2013). Taken together, these results suggest that TMS effects reported here are specific to body part images.

### 4.1 TMS over right EBA impacts self/other discrimination

One surprising result of the present study is that participants displayed a self‐advantage in the TMS runs, irrespective of the site, and delay of stimulation. This type of self‐advantage has been described with names (Alexopoulos et al., 2012), objects (Constable et al., 2018), faces (Keyes and Brady, 2010; Ma and Han, 2010; Malaspina et al., 2019), and body parts (Ferri et al., 2011; Frassinetti et al., 2010; Conson et al., 2015, 2017; Malaspina et al., 2019), in a number of different tasks. For body parts, it is less common in explicit recognition tasks (Ferri et al., 2011; Frassinetti et al., 2010; Conson et al., 2015), although it has been found when task demand increases (Conson et al., 2017). Here, although there are no significant differences between error rates between left EBA, Vertex, and noTMS, there is a non‐significant increase in errors for Other hands together with a non‐significant decrease in errors for Self Hands when TMS is applied, leading to this Self/Other difference. One explanation is that TMS induces an attentional shift or an alertness that translates into a slight self‐bias. Beyond this self‐bias, the increase in errors for Other hands was only significant for TMS over right EBA at 100–110 ms, which induced significantly more errors on other hands than any other condition.

An effect of TMS over EBA in a self/other hand discrimination paradigm has also recently been reported by De Bellis et al. (2017). These authors used repetitive three‐pulse TMS at 10 Hz, ie. 100 ms inter‐pulse intervals, with the first pulse applied 150 ms after image onset, and with the TMS output set beneath the motor threshold. While most TMS studies including ours report disruptive effects of TMS on EBA functioning, De Bellis et al. demonstrated that rTMS over rEBA facilitates the identification of others' hands. To account for this facilitation effect, the authors advocated the possibility of local cortical facilitation (Luber & Lisanby, 2014) induced by high‐frequency under‐threshold rTMS, with trains applied, while the visual processing of stimulus was still occurring. This apparent contradiction between disruptive and facilitating effects of TMS could be interpreted considering Romaiguère et al.'s results of 2005. These authors found that TMS over the sensorimotor cortex during ongoing tendon vibration could facilitate or impair kinesthetic illusion depending on whether TMS was delivered below or above motor threshold (Romaiguère et al., 2005). It is, therefore, possible that TMS over EBA would facilitate or impair EBA functioning depending on whether TMS output is above motor threshold as in the present study, or below the motor threshold as in De Bellis et al.'s study.

In addition, De Bellis et al.'s paradigm included both an explicit (visual recognition of self/others' hand images) and an implicit (laterality judgment on self and others' hand images) recognition tasks, with images being presented until participants responded (De Bellis et al., 2017). Interestingly, although TMS effects were evidenced in the implicit tasks, no difference was found in the explicit recognition task, which had a similar design to ours. One explanation for this difference is that De Bellis et al. used averaged coordinates from literature to guide TMS location, while we used individual fMRI maps which might have resulted in more precise targets. Another explanation for these divergent findings might be the difference in timing, as suggested by early works on the effects of TMS on visual processing. Indeed, pioneering works found a variety of timing relative to visual stimulus onset at which TMS over primary visual cortex was most effective at disrupting visual perception, ranging from 20 ms before to 150 ms after visual stimulus onset (Amassian et al., 1989; Beckers & Zeki, 1995; Corthout et al., 2003; Hotson et al., 1994; Lamme & Roelfsema, 2000). Taken together, these studies have demonstrated several temporal windows at which TMS pulses can induce visual suppression, reflecting perhaps multiple feedforward‐feedback loops (Corthout et al., 2003; Juan & Walsh, 2003).

The last explanation of our divergent findings with De Bellis et al. (2017) is the mechanism by which TMS disrupts processing which competes or distracts from task performance (Luber & Lisanby, 2014). In particular “addition by subtraction” mechanisms suggest a competition among the visual cortices which process different properties of incoming stimuli in parallel. For example, Walsh et al. (1998) stimulated visual motion area V5 during different visual search tasks and found that TMS could shorten or lengthen reaction times depending on whether the movement was relevant for the task. Notably, the critical time for TMS‐induced disruption varied for the different tasks between 0 and 250 ms (Walsh et al., 1998).

Taken together these results may explain why TMS applied during implicit and explicit recognition tasks has distinctive effects, yet both ours and De Bellis' studies highlight the importance of early activation in rEBA for body identification. TMS over the right EBA might disrupt the early processing of visual signals that would be important in an explicit recognition task such as ours, leading to decreased response speed and accuracy.

### 4.2 TMS impacts hand recognition processing in early time windows

In our experiment, double‐pulse TMS was applied 40/50 ms or 100/110 ms following stimulus presentation, according to a previous study evidencing two distinct time windows of EBA activity (Pitcher et al., 2012). These authors delivered double‐pulse TMS over the rEBA and the right occipital face area (rOFA) at different latencies after stimulus onset, while participants performed delayed match‐to‐sample tasks on body and face stimuli and found that only TMS delivered at 100/110 ms had category‐specific effects (bodies at rEBA and faces at rOFA) in contrast to TMS applied at 40/50 ms. It was suggested that the early and late windows could correspond to the first sweep of information into the visual cortex and to re‐entrant feedback processing from remote higher cortical areas that enhance this initial neural representation, respectively. This is compatible with our finding that TMS at both time windows delay responses, but that only TMS at 100–110 alters performance by increasing error rates on Other hands. In addition to Pitcher et al.'s (2012) work, category‐specific responses within the first 100 ms have been reported for different categories of stimuli (Liu & Ioannides, 2010; Meeren et al., 2008). Meeren et al. found category‐specific responses to human faces and bodies between 70 and 100 ms after stimulus onset (Meeren et al., 2008). Single neuron recordings in the inferior temporal cortex of monkeys have shown that the earliest part of the responses already carried information about global category (human face, monkey face or geometric shape), while specific information about expressions or identity started on average 51 ms later (Sugase et al., 1999). It is thus possible that the first sweep of information already carries information about the global category (i.e. Body or body parts) but fine‐grained analysis that allows distinction between the different body parts (including faces) starts later. This would explain why TMS applied over the EBAs at 40–50 ms delays the response but does not alter it, while TMS applied over EBA at 100–110 ms both delays and alters the response.

### 4.3 EBA is part of a network involved in self/other distinction

Although our findings suggest that rEBA contributes to identity processing, we provide no evidence that it directly processes self/other distinction. Indeed, Downing and Peelen (2011) advocated that EBA is not involved in any high‐level functions such as distinguishing body identity, but only encode fine details about visually perceived bodies. They nevertheless do not exclude the possibility that inputs from EBA to the putative higher‐order areas, as well as feedback from the latter areas to the former, are essential for person identification from bodily appearance and related cognitive abilities. They suggested that EBA provides specialized information about the form and configuration of bodies to other regions that extract and make explicit the meaningful signals of the body, making possible the identity discrimination (Downing & Peelen, 2011). Thus, EBA participates in the early phase of the visual processing of the presented body part but ownership distinction is likely processed upstream, in remote high‐order cortical areas associated with self/other distinction. In support of this view, both functional MRI experiments (Ehrsson et al., 2004, 2005; Limanowski & Blankenburg, 2016) and lesion network‐symptom‐mapping in brain‐damaged participants (Martinaud et al., 2017; Wawrzyniak et al., 2018) have associated the rubber hand illusion (an experimental protocol manipulating the sense of ownership with an artificial hand) with activity in a set of regions including the right temporoparietal junction, the right anterior insula, and right inferior frontal gyrus. Similarly, self‐face recognition has been linked to a mostly right hemisphere network (Keenan et al., 2001; Sugiura et al., 2006, 2008), that has also been found to be active in self‐body illusions leading to the proposition that it underlies self‐body recognition as well (Morita et al., 2017, 2020). On the other hand, left EBA has been shown to be active in relation to action semantics (Romaiguère et al., 2014) or during body‐part metaphor comprehension (Lacey et al., 2017). Both studies showed that the left occipito‐temporal area responding to action or metaphor comprehension was functionally connected to language processing areas in the left hemisphere known to process semantics (Lacey et al., 2017; Romaiguère et al., 2014). Lacey et al., (2017) showed that during body‐part metaphor comprehension, activity in left EBA was actually driven by that in Broca's area and in the left superior temporal gyrus. This suggests that lateralized activity in EBA could reflect the hemispheric lateralization of the brain processes involved in the task at hand. EBA would provide information about the form and configuration of bodies to higher order areas. These higher order areas would then send feedbacks to EBA. Depending on the task, these feedbacks might be strongly lateralized, and thus have a stronger modulating effect on ipsilateral EBA.

In sum, that TMS over right EBA alters self/other discrimination does not imply that right EBA has the intrinsic ability to discriminate between self and other bodies. EBA could act as an entry into larger networks underlying self/other discrimination (Downing & Peelen, 2011; Jeannerod, 2004; Kaneko et al., 2015). Disrupting EBA function during the early stage of visual discrimination would disrupt the whole network resulting in delayed/poorer discrimination.

### 4.4 Limitations of the present study

A limitation of this study is that it included only 12 participants. We acknowledge this number might seem low. However, in a study of functional accuracy of TMS in cognitive studies, Sack et al. (2009) showed that when using functional MRI guided neuronavigation five participants were sufficient to reveal a significant effect, while other guidance technics required more participants (9 for structural MRI guided neuronavigation and 13 for group Talairach coordinates). Moreover, numerous studies of the effects of TMS over various cognitive processes have shown significant effects for participants numbers between 10 and 15 (for example, Gandolfo & Downing, 2019; Luber et al., 2020; Pisoni et al., 2018; St Germain et al., 2020; van Koningsbruggen et al., 2013). We, therefore, feel that, although 12 participants is a lower number than we initially planned for, it is nonetheless enough to support the findings in this study. For the sake of future studies, we performed a power analysis using the Statistica Power analysis toolbox. Given the effect sizes observed in the present study, future studies using the same design and tasks would require at least nine participants.

Another limitation is that we chose not to counterbalance all conditions. We cannot rule out that order effects (practice/learning, high/low attention, fatigue…) might have occurred during the course of the experiment. It is, however, rather unlikely that order effects could account for the effects we report here. Indeed, there was no statistical difference between TMS over the vertex (always last) and noTMS (always first), whether on reaction times or on error rates. More notably, order effects could not account for the different effects of TMS over right and left EBAs, as the order of these conditions was counterbalanced.

Finally, we only tested right‐handed participants. There is evidence that lateralization in the lateral occipito‐temporal cortex depends on hand dominance (Willems et al., 2010), so the results reported here only pertain to right‐handers.

## 5 CONCLUSIONS

This study emphasizes the usefulness of TMS to explore visual cognition, but also the importance to control the site of stimulation using fMRI localizer, as well as the intensity and timing of stimulation. Indeed, the present study provides evidence that EBA in the right hemisphere participates in identity processing. In a previous paper, we proposed that both right and left EBA provide input into the motor system, although with distinct roles in action representation, right EBA providing input to a system involved in the processing of actions with the aim to disentangling those produced by oneself from others and experience the sense of agency (Romaiguère et al., 2014). The present study partly supports this proposition by providing evidence that right EBA does participates in self/other discrimination.

## Conflicts of interest

The authors have no conflicts of interest

## References

[phy214711-bib-0001] Abler, B. , Walter, H. , Wunderlich, A. , Grothe, J. O. , Schönfeldt‐Lecuona, C. , Spitzer, M. , & Herwig, U. (2005). Side effects of transcranial magnetic stimulation biased task performance in a cognitive neuroscience study. Brain Topography, 17(4), 193–196. 16110769 10.1007/s10548-005-6028-y

[phy214711-bib-0077] Alexopoulos, T. , Muller, D. , Ric, F. , & Marendaz, C. (2012). I, me, mine: Automatic attentional capture by self‐related stimuli. European Journal of Social Psychology, 42, 770–779.

[phy214711-bib-0002] Amassian, V. E. , Cracco, R. Q. , Maccabee, P. J. , Cracco, J. B. , Rudell, A. , & Eberle, L. (1989). Suppression of visual perception by magnetic coil stimulation of human occipital cortex. Electroencephalography and Clinical Neurophysiology/Evoked Potentials Section, 74(6), 458–462. 10.1016/0168-5597(89)90036-1 2480226

[phy214711-bib-0003] Astafiev, S. V. , Stanley, C. M. , Shulman, G. L. , & Corbetta, M. (2004). Extrastriate body area in human occipital cortex responds to the performance of motor actions. Nature Neuroscience, 7(5), 542–548. 15107859 10.1038/nn1241

[phy214711-bib-0004] Beck, B. , Bertini, C. , Haggard, P. , & Làdavas, E. (2015). Dissociable routes for personal and interpersonal visual enhancement of touch. Cortex, 73, 289–297. 26520680 10.1016/j.cortex.2015.09.008

[phy214711-bib-0005] Beckers, G. , & Zeki, S. (1995). The consequences of inactivating areas V1 and V5 on visual motion perception. Brain, 118(1), 49–60. 7895014 10.1093/brain/118.1.49

[phy214711-bib-0006] Bracci, S. , Ietswaart, M. , Peelen, M. V. , & Cavina‐Pratesi, C. (2010). Dissociable neural responses to hands and non‐hand body parts in human left extrastriate visual cortex. Journal of Neurophysiology, 103, 3389–3397. 20393066 10.1152/jn.00215.2010 PMC2888254

[phy214711-bib-0007] Chan, A. W. , Peelen, M. V. , & Downing, P. E. (2004). The effect of viewpoint on body representation in the extrastriate body area. NeuroReport, 15(15), 2407–2410. 15640765 10.1097/00001756-200410250-00021

[phy214711-bib-0081] Conson, M. , Errico, D. , Mazzarella, E. , De Bellis, F. , Grossi, D , & Trojano, L. (2015). Impact of body posture on laterality judgement and explicit recognition tasks performed on self and others’ hands. Experimental Brain Research, 233, 1331–1338. 25633320 10.1007/s00221-015-4210-3

[phy214711-bib-0078] Conson, M , Volpicella, F. , De Bellis, F. , Orefice, A. , & Trojano, L. (2017). “Like the palm of my hands”: Motor imagery enhances implicit and explicit visual recognition of one's own hands. Acta Psychologica, 180, 98–104. 28926731 10.1016/j.actpsy.2017.09.006

[phy214711-bib-0073] Constable, M. , Welsh, T. N. , Pratt, J. , & Huffman, G. (2018). I before U: Temporal order judgements reveal bias for self‐owned objects. Quaterly Journal of Experimental Psychology, 2006(4), 17470218187620. 10.1177/1747021818762010 29431023

[phy214711-bib-0008] Corthout, E. , Hallett, M. , & Cowey, A. (2003). Interference with vision by TMS over the occipital pole: A fourth period. NeuroReport, 14(4), 651–655. 12657905 10.1097/00001756-200303240-00026

[phy214711-bib-0009] De Bellis, F. , Trojano, L. , Errico, D. , Grossi, D. , & Conson, M. (2017). Whose hand is this? Differential responses of right and left extrastriate body areas to visual images of self and others' hands. Cognitive, Affective, and Behavioral Neuroscience, 17(4), 826–837. 10.3758/s13415-017-0514-z 28536919

[phy214711-bib-0010] Di Vita, A. , Boccia, M. , Palermo, L. , & Guariglia, C. (2016). To move or not to move, that is the question! Body schema and non‐action oriented body representations: An fMRI meta‐analytic study. Neuroscience and Biobehavioral Reviews, 68, 37–46. 27177829 10.1016/j.neubiorev.2016.05.005

[phy214711-bib-0011] Downing, P. E. , Jiang, Y. , Shuman, M. , & Kanwisher, N. (2001). A cortical area selective for visual processing of the human body. Science, 293(5539), 2470–2473. 11577239 10.1126/science.1063414

[phy214711-bib-0012] Downing, P. E. , & Peelen, M. V. (2011). The role of occipitotemporal body‐selective regions in person perception. Cognitive Neuroscience, 2(3–4), 186–203. 24168534 10.1080/17588928.2011.582945

[phy214711-bib-0013] Downing, P. E. , Peelen, M. V. , Wiggett, A. J. , & Tew, B. D. (2006). The role of the extrastriate body area in action perception. Social Neuroscience, 1(1), 52–62. 18633775 10.1080/17470910600668854

[phy214711-bib-0014] Duecker, F. , de Graaf, T. A. , Jacobs, C. , & Sack, A. T. (2013). Time‐ and task‐dependent non‐neural effects of real and sham TMS. PLoS One, 8(9), e73813. 24040080 10.1371/journal.pone.0073813 PMC3763998

[phy214711-bib-0015] Ehrsson, H. H. , Holmes, N. P. , & Passingham, R. E. (2005). Touching a rubber hand: Feeling of body ownership is associated with activity in multisensory brain areas. Journal of Neuroscience, 25(45), 10564–10573. 16280594 10.1523/JNEUROSCI.0800-05.2005 PMC1395356

[phy214711-bib-0016] Ehrsson, H. H. , Spence, C. , & Passingham, R. E. (2004). That's my hand! Activity in premotor cortex reflects feeling of ownership of a limb. Science, 305(5685), 875–877. 15232072 10.1126/science.1097011

[phy214711-bib-0017] Eickhoff, S. B. , Heim, S. , Zilles, K. , & Amunts, K. (2006). Testing anatomically specified hypotheses in functional imaging using cytoarchitectonic maps. NeuroImage, 32, 570–582. 16781166 10.1016/j.neuroimage.2006.04.204

[phy214711-bib-0018] Eickhoff, S. B. , Paus, T. , Caspers, S. , Grosbras, M.‐H. , Evans, A. C. , Zilles, K. , & Amunts, K. (2007). Assignment of functional activations to probabilistic cytoarchitectonic areas revisited. NeuroImage, 36, 511–521. 17499520 10.1016/j.neuroimage.2007.03.060

[phy214711-bib-0019] Eickhoff, S. B. , Stephan, K. E. , Mohlberg, H. , Grefkes, C. , Fink, G. R. , Amunts, K. , & Zilles, K. (2005). A new SPM toolbox for combining probabilistic cytoarchitectonic maps and functional imaging data. NeuroImage, 25, 1325–1335. 15850749 10.1016/j.neuroimage.2004.12.034

[phy214711-bib-0020] Felician, O. , Anton, J. L. , Nazarian, B. , Roth, M. , Roll, J. P. , & Romaiguère, P. (2009). Where is your shoulder? Neural correlates of localizing others' body parts. Neuropsychologia, 47(8), 1909–1916. 19428423 10.1016/j.neuropsychologia.2009.03.001

[phy214711-bib-0075] Ferri, F. , Frassinetti, F. , Costantini, M. , & Gallese, V. (2011). Motor simulation and the bodily self. Plos One, 6(3), e17927. 21464959 10.1371/journal.pone.0017927 PMC3064658

[phy214711-bib-0076] Frassinetti, F. , Maini, M. , Benassi, M. , Avanzi, S. , Cantagallo, A. , & Farnè, A. (2010). Selective impairment of self body‐parts processing in right brain‐damaged patients. Cortex, 46, 322–328. 19482271 10.1016/j.cortex.2009.03.015

[phy214711-bib-0021] Gandolfo, M. , & Downing, P. E. (2019). Causal evidence for expression of perceptual expectations in category‐selective extrastriate regions. Current Biology, 29, 2496–2500. 31327721 10.1016/j.cub.2019.06.024

[phy214711-bib-0022] Hodzic, A. , Kaas, A. , Muckli, L. , Stirn, A. , & Singer, W. (2009). Distinct cortical networks for the detection and identification of human body. NeuroImage, 45(4), 1264–1271. 19349239 10.1016/j.neuroimage.2009.01.027

[phy214711-bib-0023] Hodzic, A. , Muckli, L. , Singer, W. , & Stirn, A. (2009). Cortical responses to self and others. Human Brain Mapping, 30(3), 951–962. 18381769 10.1002/hbm.20558 PMC6870742

[phy214711-bib-0024] Holmes, N. P. , & Metteyard, L. (2018). Subjective discomfort of TMS predicts reaction times differences in published studies. Frontiers in Psychology, 9, 1989. 30405482 10.3389/fpsyg.2018.01989 PMC6200894

[phy214711-bib-0025] Hotson, J. , Braun, D. , Herzberg, W. , & Boman, D. (1994). Transcranial magnetic stimulation of extrastriate cortex degrades human motion direction discrimination. Vision Research, 34(16), 2115–2123. 7941409 10.1016/0042-6989(94)90321-2

[phy214711-bib-0026] Jeannerod, M. (2004). Visual and action cues contribute to the self–other distinction. Nature Neuroscience, 7(5), 422–423. 15114350 10.1038/nn0504-422

[phy214711-bib-0027] Juan, C. H. , & Walsh, V. (2003). Feedback to V1: A reverse hierarchy in vision. Experimental Brain Research, 150(2), 259–263. 12682809 10.1007/s00221-003-1478-5

[phy214711-bib-0028] Kaneko, F. , Blanchard, C. , Lebar, N. , Nazarian, B. , Kavounoudias, A. , & Romaiguère, P. (2015). Brain regions associated to a kinesthetic illusion evoked by watching a video of one's own moving hand. PLoS One, 10(8), e0131970. 26287488 10.1371/journal.pone.0131970 PMC4544853

[phy214711-bib-0029] Keenan, J. P. , Nelson, A. , O'Connor, M. , & Pascual‐Leone, A. (2001). Self‐recognition and the right hemisphere. Nature, 409, 305. 11201730 10.1038/35053167

[phy214711-bib-0079] Keyes, H. , & Brady, N. (2010). Self‐face recognition is characterized by “bilateral gain” and by faster, more acurate performance which persists when faces are inverted. Quaterly Journal of Experimental Psychology, 63(5), 840–847. 10.1080/17470211003611264 20198537

[phy214711-bib-0080] Kühn, S. , Keizer, A. , Rombouts, S. A. R. B. , & Hommel, B. (2011). The functional and neural mechanism of action preparation: Roles of EBA and FFA in voluntary action control. Journal of Cognitive Neuroscience, 23(1), 214–220. 20044885 10.1162/jocn.2010.21418

[phy214711-bib-0030] Lacey, S. , Stilla, R. , Deshpande, G. , Zhao, S. , Stephens, C. , McCormick, K. , Kemmerer, D. , & Sathian, K. (2017). Engagement of left extrastriate body area during body‐part metaphor comprehension. Brain & Language, 166, 1–18. 27951437 10.1016/j.bandl.2016.11.004

[phy214711-bib-0031] Lamme, V. A. , & Roelfsema, P. R. (2000). The distinct modes of vision offered by feedforward and recurrent processing. Trends in Neurosciences, 23(11), 571–579. 11074267 10.1016/s0166-2236(00)01657-x

[phy214711-bib-0032] Limanowski, J. , & Blankenburg, F. (2016). Integration of visual and proprioceptive limb position information in human posterior parietal, premotor, and extrastriate cortex. Journal of Neuroscience, 36(9), 2582–2589. 26937000 10.1523/JNEUROSCI.3987-15.2016 PMC6604875

[phy214711-bib-0033] Liu, L. , & Ioannides, A. A. (2010). Emotion separation is completed early and it depends on visual field presentation. PLoS One, 5(3), e9790. 20339549 10.1371/journal.pone.0009790 PMC2842434

[phy214711-bib-0034] Luber, B. , Jangraw, D. C. , Appelbaum, G. , Harrison, A. , Hilbig, S. , Beynel, L. , Jones, T. , Sajda, P. , & Lisanby, S. H. (2020). Using transcranial magnetic stimulation to test a network model of perceptual decision making in the human brain. Frontiers in Human Neuroscience, 14, 4. 10.3389/fnhum.2020.00004. 32038206 PMC6993579

[phy214711-bib-0035] Luber, B. , & Lisanby, S. H. (2014). Enhancement of human cognitive performance using transcranial magnetic stimulation (TMS). NeuroImage, 85, 961–970. 23770409 10.1016/j.neuroimage.2013.06.007 PMC4083569

[phy214711-bib-0072] Ma, Y. , & Han, S. (2010). Why we respond faster to the self than to others? An implicit positive association theory of self‐advantage during implicit face recognition. Journal of Experimental Psychology, 63(5), 840–847. 20515192 10.1037/a0015797

[phy214711-bib-0074] Malaspina, M. , Albonico, A. , & Daini, R. (2019). Self‐face and self‐body advantages in congenital prosopagnosia: Evidence for a common mechanism. Experimental Brain Research, 237, 673–686. 30542755 10.1007/s00221-018-5452-7

[phy214711-bib-0036] Martinaud, O. , Besharati, S. , Jenkinson, P. M. , & Fotopoulou, A. (2017). Ownership illusions in patients with body delusions: Different neural profiles of visual capture and disownership. Cortex, 87, 174–185. 27839786 10.1016/j.cortex.2016.09.025 PMC5312675

[phy214711-bib-0037] Meeren, H. K. , Hadjikhani, N. , Ahlfors, S. P. , Hämäläinen, M. S. , & de Gelder, B. (2008). Early category‐specific cortical activation revealed by visual stimulus inversion. PLoS One, 3(10), e3503. 18946504 10.1371/journal.pone.0003503 PMC2566817

[phy214711-bib-0038] Meteyard, L. , & Holmes, N. P. (2018). TMS Smart – Scalp mapping of annoyance ratings and twitches caused by transcranial magnetic stimulation. Journal of Neuroscience Methods, 299, 34–44. 29471064 10.1016/j.jneumeth.2018.02.008

[phy214711-bib-0039] Morita, T. , Asada, M. , & Naito, E. (2020). Right‐hemispheric dominance in self‐body recognition is altered in left‐handed individuals. Neuroscience, 425, 68–89. 31809726 10.1016/j.neuroscience.2019.10.056

[phy214711-bib-0040] Morita, T. , Saito, D. N. , Ban, M. , Shimada, K. , Okamoto, Y. , Kosaka, H. , Okazawa, H. , Asada, M. , & Naito, E. (2017). Self‐face recognition shares brain regions active during proprioceptive illusion in the right inferior fronto‐parietal superior longitudinal fasciculus III network. Neuroscience, 348, 288–301. 28238849 10.1016/j.neuroscience.2017.02.031

[phy214711-bib-0041] Myers, A. , & Sowden, P. T. (2008). Your hand or mine? The extrastriate body area. NeuroImage, 42(4), 1669–1677. 18586108 10.1016/j.neuroimage.2008.05.045

[phy214711-bib-0042] Orlov, T. , Makin, T. R. , & Zohary, E. (2010). Topographic representation of the human body in the occipitotemporal cortex. Neuron, 68(3), 586–600. 21040856 10.1016/j.neuron.2010.09.032

[phy214711-bib-0043] Peelen, M. V. , & Downing, P. E. (2007). The neural basis of visual body perception. Nature Reviews Neuroscience, 8(8), 636–648. 17643089 10.1038/nrn2195

[phy214711-bib-0044] Pinsk, M. A. , Arcaro, M. , Weiner, K. S. , Kalkus, J. F. , Inati, S. J. , Gross, C. G. , & Kastner, S. (2009). Neural representations of faces and body parts in macaque and human cortex: A comparative FMRI study. Journal of Neurophysiology, 101(5), 2581–2600. 19225169 10.1152/jn.91198.2008 PMC2681436

[phy214711-bib-0045] Pisoni, A. , Romero Lauro, L. J. , Vergallito, A. , Maddaluno, O. , & Bolognini, N. (2018). *NeuroImage*, 178, 475–484. 10.1016/j.neuroimage.2018.05.078 29860085

[phy214711-bib-0046] Pitcher, D. , Charles, L. , Devlin, J. T. , Walsh, V. , & Duchaine, B. (2009). Triple dissociation of faces, bodies, and objects in extrastriate cortex. Current Biology, 19(4), 319–324. 19200723 10.1016/j.cub.2009.01.007

[phy214711-bib-0047] Pitcher, D. , Goldhaber, T. , Duchaine, B. , Walsh, V. , & Kanwisher, N. (2012). Two critical and functionally distinct stages of face and body perception. Journal of Neuroscience, 32(45), 15877–15885. 23136426 10.1523/JNEUROSCI.2624-12.2012 PMC3752141

[phy214711-bib-0048] Romaiguère, P. , Calvin, S. , & Roll, J. P. (2005). Transcranial magnetic stimulation of the sensorimotor cortex alters kinaesthesia. NeuroReport, 16(7), 693–697. 15858408 10.1097/00001756-200505120-00008

[phy214711-bib-0049] Romaiguère, P. , Nazarian, B. , Roth, M. , Anton, J. L. , & Felician, O. (2014). Lateral occipitotemporal cortex and action representation. Neuropsychologia, 56, 167–177. 24467888 10.1016/j.neuropsychologia.2014.01.006

[phy214711-bib-0050] Rossi, S. , Hallett, M. , Rossini, P. M. , & Pascual‐Leone, A. (2009). Safety, ethical considerations, and application guidelines for the use of transcranial magnetic stimulation in clinical practice and research. Clinical Neurophysiology, 120(12), 2008–2039. 19833552 10.1016/j.clinph.2009.08.016 PMC3260536

[phy214711-bib-0051] Sack, A. T. , Cohen Kadosh, R. , Schuhmann, T. , Moerel, M. , Walsh, V. , & Goebel, R. (2009). Optimizing functional accuracy of TMS in cognitive studies: A comparison of methods. Journal of Cognitive Neuroscience, 21(2), 207–221. 18823235 10.1162/jocn.2009.21126

[phy214711-bib-0052] Saxe, R. , Jamal, N. , & Powell, L. (2006). My body or yours? The effect of visual perspective on cortical body representations. Cerebral Cortex, 16(2), 178–182. 15858162 10.1093/cercor/bhi095

[phy214711-bib-0053] Schwarzlose, R. F. , Swisher, J. D. , Dang, S. , & Kanwisher, N. (2008). The distribution of category and location information across object‐selective regions in human visual cortex. Proceedings of the National Academy of Sciences of the United States of America, 105(11), 4447–4452. 18326624 10.1073/pnas.0800431105 PMC2393746

[phy214711-bib-0054] Silvanto, J. , Lavie, N. , & Walsh, V. (2005). Double dissociation of V1 and V5/MT activity in visual awareness. Cerebral Cortex, 15(11), 1736–1741. 15703247 10.1093/cercor/bhi050

[phy214711-bib-0055] Spiridon, M. , Fischl, B. , & Kanwisher, N. (2006). Location and spatial profile of category‐specific regions in human extrastriate cortex. Human Brain Mapping, 27(1), 77–89. 15966002 10.1002/hbm.20169 PMC3264054

[phy214711-bib-0056] St Germain, L. , Smith, V. , Maslovat, D. , & Carlsen, A. (2020). Increased auditory stimulus intensity results in an earlier and faster rise in corticospinal excitability. Brain Research, 1727, 146559. 31734397 10.1016/j.brainres.2019.146559

[phy214711-bib-0057] Sugase, Y. , Yamane, S. , Ueno, S. , & Kawano, K. (1999). Global and fine information coded by single neurons in the temporal visual cortex. Nature, 400, 869–872. 10476965 10.1038/23703

[phy214711-bib-0058] Sugiura, M. , Sassa, Y. , Jeong, H. , Horie, K. , Sato, S. , & Kawashima, R. (2008). Face‐specific and domain‐general characteristics of cortical responses during self‐recognition. NeuroImage, 42(1), 414–422. 18501639 10.1016/j.neuroimage.2008.03.054

[phy214711-bib-0059] Sugiura, M. , Sassa, Y. , Jeong, H. , Miura, M. , Akitsuki, Y. , Horie, K. , Sato, S. , & Kawashima, R. (2006). Multiple brain networks for visual self‐recognition with different sensitivity for motion and body part. NeuroImage, 32(4), 1905–1917. 16806977 10.1016/j.neuroimage.2006.05.026

[phy214711-bib-0060] Taylor, J. C. , Wiggett, A. J. , & Downing, P. E. (2007). Functional MRI analysis of body and body part representations in the extrastriate and fusiform body areas. Journal of Neurophysiology, 98(3), 1626–1633. 17596425 10.1152/jn.00012.2007

[phy214711-bib-0061] Tzourio‐Mazoyer, N. , Landeau, B. , Papathanassiou, D. , Crivello, F. , Etard, O. , Delcroix, N. , Mazoyer, B. , & Joliot, M. (2002). Automated anatomical labeling of activations in SPM using a macroscopic anatomical parcellation of the MNI MRI single‐subject brain. NeuroImage, 15, 273–289. 11771995 10.1006/nimg.2001.0978

[phy214711-bib-0062] Urgesi, C. , Berlucchi, G. , & Aglioti, S. M. (2004). Magnetic stimulation of extrastriate body area impairs visual processing of nonfacial body parts. Current Biology, 14(23), 2130–2134. 15589156 10.1016/j.cub.2004.11.031

[phy214711-bib-0063] Urgesi, C. , Calvo‐Merino, B. , Haggard, P. , & Aglioti, S. M. (2007). Transcranial magnetic stimulation reveals two cortical pathways for visual body processing. Journal of Neuroscience, 27(30), 8023–8030. 17652592 10.1523/JNEUROSCI.0789-07.2007 PMC6672742

[phy214711-bib-0064] Van Koningsbruggen, M. G. , Peelen, M. V. , & Downing, P. E. (2013). A causal role for the extrastriate body area in detecting people in real‐world scenes. The Journal of Neuroscience, 33(16), 7003–7010. 23595757 10.1523/JNEUROSCI.2853-12.2013 PMC6618865

[phy214711-bib-0065] Vocks, S. , Busch, M. , Grönemeyer, D. , Schulte, D. , Herpertz, S. , & Suchan, B. (2010). Differential neuronal responses to the self and others in the extrastriate body area and the fusiform body area. Cognitive, Affective, and Behavioral Neuroscience, 10(3), 422–429. 10.3758/CABN.10.3.422 20805543

[phy214711-bib-0066] Walsh, V. , Ellison, A. , Battelli, L. , & Cowey, A. (1998). Task–specific impairments and enhancements induced by magnetic stimulation of human visual area V5. Proceedings of the Royal Society of London B: Biological Sciences, 265(1395), 537–543. 10.1098/rspb.1998.0328 PMC1688918 9569672

[phy214711-bib-0067] Walsh, V. , & Pascual‐Leone, A. (2003). Transcranial magnetic stimulation: A neurochronometrics of mind. MIT press.

[phy214711-bib-0068] Wassermann, E. M. (1998). Risk and safety of repetitive transcranial magnetic stimulation: Report and suggested guidelines from the international workshop on the safety of repetitive transcranial magnetic stimulation, June 5–7, 1996. Electroencephalography and Clinical Neurophysiology/Evoked Potentials Section, 108(1), 1–16. 10.1016/s0168-5597(97)00096-8 9474057

[phy214711-bib-0069] Wawrzyniak, M. , Klingbeil, J. , Zeller, D. , Saur, D. , & Classen, J. (2018). The neuronal network involved in self‐attribution of an artificial hand: A lesion network‐symptom‐mapping study. NeuroImage, 166, 317–324. 29122723 10.1016/j.neuroimage.2017.11.011

[phy214711-bib-0070] Willems, R. M. , Peelen, M. V. , & Hagoort, P. (2010). Cerebral lateralization of face‐selective and body‐selective visual areas depends on handedness. Cerebral Cortex, 20, 1719–1725. 19889713 10.1093/cercor/bhp234

[phy214711-bib-0071] Yousry, T. A. , Schmid, U. D. , Alkadhi, H. , Schmidt, D. , Peraud, A. , Buettner, A. , & Winkler, P. (1997). Localization of the motor hand area to a knob on the precentral gyrus. A new landmark. Brain, 120(1), 141–157. 9055804 10.1093/brain/120.1.141

